# Research priorities for the COVID‐19 pandemic and beyond: A call to action for psychological science

**DOI:** 10.1111/bjop.12468

**Published:** 2020-07-19

**Authors:** Daryl B. O'Connor, John P. Aggleton, Bhismadev Chakrabarti, Cary L. Cooper, Cathy Creswell, Sandra Dunsmuir, Susan T. Fiske, Susan Gathercole, Brendan Gough, Jane L. Ireland, Marc V. Jones, Adam Jowett, Carolyn Kagan, Maria Karanika‐Murray, Linda K. Kaye, Veena Kumari, Stephan Lewandowsky, Stafford Lightman, Debra Malpass, Elizabeth Meins, B. Paul Morgan, Lisa J. Morrison Coulthard, Stephen D. Reicher, Daniel L. Schacter, Susan M. Sherman, Victoria Simms, Antony Williams, Til Wykes, Christopher J. Armitage

**Affiliations:** ^1^ School of Psychology University of Leeds UK; ^2^ School of Psychology Cardiff University UK; ^3^ School of Psychology & Clinical Language Sciences University of Reading UK; ^4^ Alliance Manchester Business School University of Manchester UK; ^5^ Departments of Experimental Psychology and Psychiatry University of Oxford UK; ^6^ Educational Psychology Group Division of Psychology and Language Sciences University College London UK; ^7^ Department of Psychology and School of International and Public Affairs Princeton University New Jersey USA; ^8^ Department of Psychiatry University of Cambridge UK; ^9^ Leeds School of Social Sciences Leeds Beckett University UK; ^10^ School of Psychology University of Central Lancashire Preston UK; ^11^ Mersey Care NHS Foundation Trust Liverpool UK; ^12^ Department of Psychology Manchester Metropolitan University UK; ^13^ School of Psychological Social & Behavioural Sciences Coventry University UK; ^14^ School of Psychology Manchester Metropolitan University UK; ^15^ Department of Psychology Nottingham Trent University UK; ^16^ Edge Hill University Lancashire UK; ^17^ Centre for Cognitive Neuroscience College of Health and Life Sciences Brunel University London UK; ^18^ School of Psychological Science and Cabot Institute University of Bristol UK; ^19^ Translational Health Sciences, Bristol Medical School University of Bristol UK; ^20^ British Psychological Society Leicester UK; ^21^ Department of Psychology University of York UK; ^22^ Systems Immunity URI Cardiff School of Medicine Cardiff University UK; ^23^ School of Psychology and Neuroscience University of St Andrews UK; ^24^ Department of Psychology Harvard University Cambridge Massachusetts USA; ^25^ School of Psychology Keele University UK; ^26^ School of Psychology Ulster University Coleraine UK; ^27^ School of Education University of Sheffield UK; ^28^ Institute of Psychiatry, Psychology and Neuroscience, King's College London UK; ^29^ South London and Maudsley NHS Foundation Trust UK; ^30^ Manchester Centre for Health Psychology School of Health Sciences University of Manchester UK; ^31^ Manchester University NHS Foundation Trust Manchester Academic Health Science Centre UK

**Keywords:** behaviour change, children, COVID‐19, education, families, health, human development, mental health, neuroscience, pandemic, psychological science, psychology, school, stress, trauma, work

## Abstract

The severe acute respiratory syndrome coronavirus‐2 (SARS‐CoV‐2) that has caused the coronavirus disease 2019 (COVID‐19) pandemic represents the greatest international biopsychosocial emergency the world has faced for a century, and psychological science has an integral role to offer in helping societies recover. The aim of this paper is to set out the shorter‐ and longer‐term priorities for research in psychological science that will (a) frame the breadth and scope of potential contributions from across the discipline; (b) enable researchers to focus their resources on gaps in knowledge; and (c) help funders and policymakers make informed decisions about future research priorities in order to best meet the needs of societies as they emerge from the acute phase of the pandemic. The research priorities were informed by an expert panel convened by the British Psychological Society that reflects the breadth of the discipline; a wider advisory panel with international input; and a survey of 539 psychological scientists conducted early in May 2020. The most pressing need is to research the negative biopsychosocial impacts of the COVID‐19 pandemic to facilitate immediate and longer‐term recovery, not only in relation to mental health, but also in relation to behaviour change and adherence, work, education, children and families, physical health and the brain, and social cohesion and connectedness. We call on psychological scientists to work collaboratively with other scientists and stakeholders, establish consortia, and develop innovative research methods while maintaining high‐quality, open, and rigorous research standards.

## Background

The global impact of the coronavirus disease 2019 (COVID‐19) is unprecedented. By the 20 June 2020, in excess of 8 million cases of COVID‐19 worldwide had been confirmed and COVID‐19‐related deaths were close to half a million. However, its impact should not only be measured in terms of biological outcomes, but also in terms of its economic, health, psychological, and social consequences. The COVID‐19 pandemic is unique with respect to the ongoing risks associated with the large numbers of infected people who remain asymptomatic, the impacts of the countermeasures on societies, the likelihood of second or third waves, and the attention it has received due to its global reach (particularly in high‐income countries). The effects of the COVID‐19 pandemic will likely shape human behaviour in perpetuity. Psychological science is uniquely placed to help mitigate the many shorter‐ and longer‐term consequences of the pandemic and to help with recovery and adjustment to daily life.

The immediate research response to COVID‐19 was rightly to focus resources on the transmission of COVID‐19, identify biologics with which to treat those infected with the virus, and develop vaccines to protect populations. However, biomedical science can only go so far in mitigating the severe negative health, economic, psychological, and social impacts of COVID‐19. The future availability of a vaccine currently remains uncertain; therefore, the primary weapons to mitigate the pandemic are behavioural, such as encouraging people to observe government instructions, self‐isolation, quarantining, and physical distancing. Even if a vaccine becomes available, we will still require changes in behaviour to ensure its effective delivery and universal uptake, so we need to prioritize research that will make the greatest contributions to our understanding of the effects of, and recovery from, the pandemic.

The important contributions made by psychological scientists to understanding the impact of previous pandemics, including the Ebola disease outbreak, severe acute respiratory syndrome (SARS), and the Middle East respiratory syndrome (MERS), are well‐documented and mean we knew already a lot about public messaging and stress among frontline workers when the COVID‐19 outbreak began (e.g., Brooks *et al*., 2020, Holmes *et al*., 2020; Rubin, Potts, & Michie, 2010; Tam, Pang, Lam, & Chiu, 2004; Thompson, Garfin, Holman, & Silver, 2017; Wu *et al*., 2009). However, the unique features of COVID‐19, including its virulence, the large proportions of people who remain asymptomatic but may still spread the virus (Centre for Evidence‐based Medicine, 2020), the stringent lockdown procedures imposed at pace on whole societies, and its global reach mean there is an urgent and ongoing need for social science research (World Health Organisation, 2020).

The collective and individual responses to severe acute respiratory syndrome coronavirus‐2 (SARS‐CoV‐2) and to the introduction of measures to counter it have fundamentally changed how societies function, affecting how we work, educate, parent, socialize, shop, communicate, and travel. It has led to bereavements at scale, as well as frontline workers being exposed to alarming levels of stress (e.g., British Medical Association, 2020; Greenberg, Docherty, Gnanapragasam, & Wessely, 2020). There have additionally been nationwide ‘lockdowns’ comprising physical distancing, quarantines, and isolation with the associated effects on loneliness, forced remote working, and homeschooling (e.g., Hoffart, Johnson, & Ebrahimi, 2020; Holmes *et al*., 2020; Lee, 2020). However, as well as having adverse psychological effects, the measures introduced to fight the pandemic may have led to positive social and behavioural changes. Most obvious are the remarkable levels of compassion and support that have developed among neighbours and within communities as well as positive changes in behaviours such as hand hygiene, homeschooling, and physical activity. Therefore, in addition to mitigating the negative effects of the pandemic, it is important to understand how any positive effects can be maintained as restrictions ease.

There are, and will undoubtedly continue to be, inequalities in the effects of the pandemic and its aftermath; recognizing these vulnerability and resilience factors will be key to understanding how the current situation can inform and prepare us for dealing with future crises. Of course, while we, as psychological scientists, are interested in the general effects of the pandemic, we are acutely aware of the fact that these effects disproportionately impact on different groups (Box 1). The issue of inequality is of central importance and runs through the research priorities that we describe below and it is surely not a coincidence that the murder of George Floyd during a global pandemic prompted a global civil rights movement drawing attention to inequalities.

Box 1 **Inequalities**

A picture is emerging of COVID‐19 not as a single pandemic, but multiple parallel pandemics with some people facing numerous severe challenges and others experiencing few or none (Williamson *et al*., 2020). For those most vulnerable groups, the social, economic, and consequent psychological challenges of the pandemic are likely to be far‐reaching and sustained. A clear priority for psychological scientists is to understand how best to help those in need and to consider the following factors in their research efforts.

**Ethnicity**

In Western Europe and the United States, the death rate among people with black, Asian, and minority ethnic backgrounds is substantially higher than that of the general population. It is not known what is causing the disproportionate impact nor how it can be mitigated. Psychological science is in a good position to explore the biopsychosocial antecedents and consequences of having a black, Asian, or minority ethnic background in the context of COVID‐19.

**Socio‐economic status**

Individuals living in poverty face disproportionate challenges in relation to education, work, income, housing, and physical and mental health. For these most vulnerable groups, the social, economic, and consequent psychological challenges of the pandemic are likely to be far‐reaching and sustained. Moreover, an impending financial crisis means that people who have never before experienced hardships may suddenly find themselves in precarious circumstances.

**Health**

A quarter of people in the UK experience mental health problems every year, with particularly high levels in young people (Mental Health Foundation, 2020). The changed social conditions of the pandemic may increase the severity of mental health challenges, particularly when standard (face‐to‐face) treatment and support are difficult to access. At the same time, pregnant women and those with existing long‐term conditions such as transplant patients, cancer patients, and chronic obstructive pulmonary disease patients have been designated ‘extremely vulnerable’ and asked to self‐isolate for long periods of time with uncertainties over access to support. Those individuals who have recovered from COVID‐19 might also have new biological vulnerabilities, uncertainty over immunity post‐COVID‐19, and risk stigma arising from infection. Individuals with disabilities, learning disabilities, special educational needs, and developmental disorders may also be more vulnerable due to the increased psychological challenges associated with shielding and self‐isolation.

**Age and sex**

The challenges generated by the pandemic vary markedly across the lifespan and will influence the nature of current and future psychological needs of different groups. Many young people have struggled with reductions in direct social contact, decreased motivation, and uncertainty caused by disrupted training and education. Adults have experienced multiple stresses as a consequence of intensified caring responsibilities, financial concerns, job uncertainty, and health conditions. For many older people, the greatest challenges have been social isolation, disruptions in access to health and social care, and coping with bereavement. In addition to the challenges surrounding age, there are emerging data to suggest that the effects of COVID‐19 may exacerbate existing inequalities for women. For example, women are more likely to be key workers and primary caregivers, thereby being exposed to higher levels of psychological and financial stress (Fawcett Society, 2020).

**Social exclusion and social support**

The COVID‐19 pandemic is likely to have had a disproportionate impact on groups with low levels of social inclusion and/or those who traditionally have declined support services, such as people living in poverty, traveller communities, and people who are homeless. Being separated from wider support networks may also be particularly difficult for those living in hostile households such as victims of domestic abuse and LGBT people living with family members who are unaccepting of their identity. Many of those detained in secure settings have been exposed to marked changes in service delivery and reduced social contact, increasing their vulnerability to the psychological effects of the pandemic. Conversely, well‐functioning social support is likely to confer resilience against the negative psychological impacts of the pandemic.

**Intersectionality**

Finally, it is important that psychological scientists consider the interconnectedness of the above factors. For example, individuals who are young and from a BAME background who are also from a less affluent socio‐economic background may be disproportionately impacted by the educational, economic, and other consequences of the measures taken to contain and recover from the pandemic. Similarly, many of the solutions to the problems posed by the pandemic involve the use of new technologies that assume the requisite skills, access to devices, and Internet connectivity meaning that the ‘digital divide’ will likely have been exacerbated by the pandemic (ONS, 2019).

In this position paper, informed by a group of experts and a survey (Box 2), we highlight the many ways in which psychological science, its methods, approaches, and interventions can be harnessed to help governments, policymakers, national health services, education sectors, and economies recover from COVID‐19 (Box 3) and other future pandemics (if they occur). Specifically, we have identified the shorter‐ and longer‐term priorities around mental health, behaviour change and adherence, work, education, children and families, physical health and the brain, and social cohesion and connectedness in order to (1) frame the breadth and scope of potential contributions from across the discipline, (2) assist psychological scientists in focusing their resources on gaps in the literature, and (3) help funders and policymakers make informed decisions about the shorter‐ and longer‐term COVID‐19 research priorities to meet the needs of societies as they emerge from the acute phase of the crisis.

Box 2 **Methodology for this position paper**

This paper outlines research priorities for psychological science for the COVID‐19 pandemic. In April 2020, the British Psychological Society convened a core group of nine experts who met regularly for 4 weeks in order to develop the research priorities. The nine experts^1^ represent broad areas of the discipline, namely biological, clinical, cognitive, developmental, educational, health, occupational, and social, and were assisted by a wider advisory group^2^ of psychological scientists (*n* = 16) drawn from a range of UK higher education institutions and areas of research expertise. We also received input from two international experts. Briefly, we used an iterative expert consensus procedure (e.g., Merry, Cooper, Soyannwo, Wilson, & Eichhorn, 2010) to elicit and distil the judgments of experts on the research priorities for psychological science. Unlike other consensus methods, which typically start with a list of priorities that are then ranked over the course of 2 or 3 meetings (e.g., Fitch, Bernstein, Aguilar, Burnand, & LaCalle, 2001; McMillan, King, & Tully, 2016), the present approach both generated and judged the priorities over 10 hour‐long face‐to‐face meetings of the core group. Consensus was achieved through discussion, and the experts were encouraged to discuss with the wider advisory group and their professional networks in between meetings.

Given the need to establish the priorities rapidly, a lengthy consultation process or an extensive review of all relevant scientific literatures was not possible. However, a brief online survey of psychological scientists was launched early in May 2020 with the aim of ensuring that the core and advisory groups had not missed any key research priorities, and to identify the highest ranked priorities in each of the broad areas of psychology to help inform the final wider‐ranging research priority domains. The online survey had two components: First, participants were asked the open‐ended question, ‘Please can you tell us what are your priorities for psychological science research in response to the COVID‐19 pandemic?’ Second, participants were asked to rank order the top five research priorities identified by the core group in each of the eight broad areas of the discipline (i.e., biological, clinical, cognitive, developmental, educational, health, occupational, social). The survey was distributed to psychologists via Heads of UK Psychology Department email lists, the social media outlets of professional psychology networks (including the British Psychological Society), and snowball email methods by the expert and advisory group members. We received replies from 539 psychological scientists representing all of the main areas of the discipline. Respondents were 75.6% female, 73.8% were aged between 31 and 60 years, and 20.1% self‐identified as being from a minority group. The highest ranked research priorities in each of the broad areas are presented in Table 1 (see Appendix for the full list of 40 priorities).

As a result of the time constraints, a detailed qualitative analysis was not possible for inclusion in this paper; nevertheless, the core group gave consideration to all of the free responses provided. Overall, there were differing degrees of specificity, and respondents provided numerous, additional, and well‐specified research questions. However, at the broadest level, respondents' priorities coalesced around the question of how do we address the negative biopsychosocial effects of the COVID‐19 pandemic? The degrees of specificity related to population (e.g., people with black, Asian, and minority ethnic backgrounds, children, people with low socio‐economic status, people living with long‐term conditions), type of intervention (e.g., service provision, environmental/social planning), methodology (e.g., qualitative, online, survey, laboratory‐based), and setting (e.g., workplace, school, prison), but there was broad agreement.

That is not to say that all research priorities were covered in the original survey. Two issues in particular stood out from the comments we received. The first was the importance of dealing with inequalities and differences between groups in the experience of the pandemic. The second was the need to address the positive as well as the negative developments coming out of the response to COVID‐19. These were both incorporated into revisions of the paper and now occupy a much more central place than before. We are thankful to all those anonymous respondents whose comments helped improve our argument. A more rigorous, thematic analysis of these data is now available (see BPS, 2020c).

The picture was very similar when respondents were asked to place research priorities identified by the expert group into rank orders. That is, broadly speaking, the priorities that received the highest rankings, irrespective of area of subdiscipline, were related to the need to address the negative biopsychosocial effects of the COVID‐19 pandemic.

**Table 1:** Highest ranked item in each broad area of psychology and its associated mean rank within that area

Area	Question	Mean rank (1 = highest 5 = lowest)	*SD*	1st rank per cent
Biological	How do we address the negative biological impacts of the COVID‐19 virus on mental health?	1.99	1.25	42.6%
Clinical	What coping mechanisms are useful in reducing mental health problems during a pandemic?	1.97	1.10	43.1%
Cognitive	What are the impacts of COVID‐19 infection, treatment, and recovery on cognition, behaviour, and the brain?	2.91	1.52	27.3%
Developmental	How has the COVID‐19 pandemic affected children's development?	2.43	1.36	35.5%
Educational	How do school closures influence educational progress, and physical and mental health outcomes for all children and young people?	2.33	1.30	36.2%
Health	How do we address the negative psychological impacts of the COVID‐19 pandemic?	2.46	1.26	28.4%
Occupational	What is the impact of remote and flexible working arrangements on employee health, mental well‐being, teamwork, performance, organizational productivity, and colleague/client relationships?	2.39	1.30	33.6%
Social	What makes people adhere to anti‐COVID measures?	2.16	1.38	48.2%

Box 3 **Psychological science: methods, approaches, and interventions to help meet the immediate and longer‐term COVID‐19 research priorities**

The future research landscape will be challenging due to the ongoing physical distancing requirements; however, psychological scientists are equipped with a broad range of methods, approaches, and interventions that will allow these research priorities to be met. Some examples are as follows:

**Online research methods**

Internet‐mediated research will be an important approach utilized by psychological scientists to collect data in the immediate post‐pandemic phase and at longer‐term follow‐ups. Internet‐mediated research can be reactive (e.g., online surveys, online interviews) and non‐reactive (e.g., data mining, observations from screen‐time apps) and can be integrated with objective assessments of behaviour as well as with biological and social markers of physical and mental health. Internet‐mediated research can also be used to run experiments with online software available such as Gorilla, PsychoPy, and e‐Prime. Recent work has summarized the range of software for building behavioural tasks, and their efficacy in being used online (Sauter, Draschkow, & Mack, 2020). Changes in the use of research methodologies may provide a catalyst for the formation of new collaborations and training to develop research skills in the psychological science community. At the same time, trust around data security and confidentiality will need to be built between researchers and the general public from whom we sample. However, in 2018, an estimated 100 million people aged 16–74 years in the European Union reported they had not used the Internet in the preceding 3 months (Eurostat, 2019), and researchers will need to think creatively about conducting research projects remotely. For example, participants can have study materials delivered by post (e.g., Salivettes for cortisol sampling or asking participants to self‐sample), replacing face‐to‐face communication with telephone and/or video calls, and the use of personal protective equipment when collecting data.

**Remotely delivered interventions**

Psychological therapies and behaviour change interventions can already be delivered remotely and evidence suggests that remote delivery does not necessarily mean inferior delivery (e.g., Irvine *et al*., 2020). Urgent research is needed to translate interventions that are typically delivered in‐person to telephone and online delivery modalities.

**Qualitative methods**

Psychologists are well‐positioned to collect valuable qualitative data concerning people's relevant experiences, perspectives, and practices associated with COVID‐19, which could inform psychology‐based interventions to improve well‐being and social cohesion. Multiple participant‐centred qualitative research methods can be rapidly deployed to elicit first‐hand accounts from members of different communities, including (online) interviews, focus groups, and qualitative questionnaires, focusing on the psychological and social impact (Jowett, 2020). Beyond the immediate term, qualitative data can be gathered longitudinally so that insights can be generated into the experiences of diverse groups over time, identifying salient crisis points and effective resolutions.

**Implementation science and psychological science**

Implementation science is a branch of psychological science that is dedicated to the uptake and use of research into clinical, educational, health care, organizational, and policy settings. Principles of implementation science can be used to help stakeholders navigate the extensive and unwieldy psychological science research literature. To inform policymakers and support professional decision‐making about implementation, psychological research needs to be disseminated in an accessible format. One example of a well‐regarded translational system is the US Institute of Education Sciences What Works Clearinghouse (https://ies.ed.gov/ncee/wwc/), which provides reviews and recommendations about evidence‐based practices for professionals working in educational settings.

**Providing expertise at pace**

An important feature of the COVID‐19 pandemic has been requested by government to provide psychological science expertise at pace. The inclination of many psychological scientists is to begin designing a new study or conducting a systematic review following Preferred Reporting Items for Systematic Reviews and Meta‐analyses (PRISMA) guidelines, but this does not meet the needs of policymakers. It would be valuable for psychological scientists providing expert advice to acquaint themselves with the terminology and procedures that are familiar to civil servants who are more likely to have use for a quick scoping review or rapid evidence assessment (Collins, Coughlin, Miller, & Kirk, 2015) rather than embarking on a time‐consuming systematic review of systematic reviews (Keyworth, Epton, Goldthorpe, Calam, & Armitage, 2020).

**Patient and public involvement in research**

There are many challenges involved with conducting COVID‐19‐related research including dealing with vulnerable groups, giving due consideration to ethical concerns, as well as issues around running studies in the light of physical distancing requirements. Therefore, having relevant patient and public involvement and including individuals with lived experience (as appropriate) in designing studies will be of paramount importance.

**Open science principles and approaches**

Psychological science has been leading the way in promoting and adopting Open Science principles and practices. Nevertheless, psychological scientists need to ensure they balance the urgency of conducting COVID‐related research (during and in the recovery period) with ensuring research quality and open research practices. Therefore, in order to help maintain quality, openness, and rigour, we urge researchers to endeavour to use registered reports, where possible (e.g., https://osf.io/rr/), or pre‐register their research hypotheses and analysis plans (e.g., https://aspredicted.org/) and make their data findable, accessible, interoperable, reusable (FAIR) recognizing the principle of ‘as open as possible; as closed as necessary’ (BPS, 2020a, 2020b; Norris & O'Connor, 2019). Moreover, we urge researchers to utilize pre‐print servers, such as PsyArXiv, in order to ensure their latest research findings are made publicly available rapidly and at no cost. We hope that openness will drive quality, but as yet there is no substitute for articles being peer‐reviewed prior to wider acceptance by the scientific community.

**Need for coordinated effort**

Psychological science has responded swiftly to the COVID‐19 pandemic, but there is a danger of duplication of efforts and participant fatigue in the proliferation of online surveys, experiments, and focus groups that have arisen. We need to harness the ongoing efforts of psychological scientists worldwide in a coordinated effort on the scale of the Large Hadron Collider (CERN, 2009) to deliver truly evidence‐based interventions to help societies emerge from the COVID‐19 pandemic. This will include cross‐cultural research to understand why mortality rates, mitigation measures, and adherence to government instructions have differed so markedly between countries. Finally, we urge researchers to register their research studies and findings on international repositories (https://osf.io/collections/coronavirus/discover).

## Research priority domains

The methodology we employed to develop the main research priority domains is described in Box 2, and the seven priority domains are outlined below and summarized in Table 2.

**Table 2:** Summary of research priority domains

**1. Groups, cohesion, and conflict**
How does collective identification impact on social responsibility and adherence to anti‐pandemic measures? How can we nurture the development and persistence of mutual aid and pro‐social behaviours? What is the relationship between group membership, connectedness, and well‐being? Under what conditions do unity and social solidarity give way to intergroup division and social conflict?
**2. Work environment and working arrangements**
What is the impact of remote and flexible working arrangements on employee health, mental well‐being, teamwork, performance, organizational productivity, and colleague/client relationships? What is the impact of social distancing in the workplace on employee health, mental well‐being, teamwork, performance, organizational productivity, and colleague/client relationships? How can organizational resilience be developed to deal with the impact of COVID‐19 whilst supporting employees and protecting jobs?
**3. Children and families**
How will the COVID‐19 pandemic affect children's development? How will the COVID‐19 pandemic affect family functioning?
**4. Educational practices**
How do school closures influence children's educational progress and well‐being? What kinds of support improve long‐term outcomes for children and young people? How can support services be effectively delivered to vulnerable children and young people, families, and schools?
**5. Mental health**
What are the immediate and longer‐term consequences of COVID‐19 for mental health outcomes? What changes in approaches resulting from the pandemic need to be harnessed for the future?
**6. Physical health and the brain**
Does COVID‐19 have neurological effects on the brain with consequences for mental health? What are the psychobiological impacts of the COVID‐19 pandemic on physical and mental health?
**7. Behaviour change and adherence**
How do we best apply existing theories and tools to promote sustained behaviour change among policymakers, key workers, and the public/patients? How do we develop new theories and tools to promote sustained behaviour change?

### Groups, cohesion, and conflict

#### How does collective identification impact on social responsibility and adherence to anti‐pandemic measures?

One of the most striking aspects of the COVID‐19 pandemic has been the importance of social psychology to the outcomes. Given the highly differentiated nature of susceptibility to the virus (Box 1), one might have expected many (especially the young and fit) to conclude that they have more to lose than gain by observing the rigours of lockdown and other preventative measures. If they had acted on such an individualistic calculus, then far more people would get infected and far more (especially the old and infirm) would die. However, on the whole, people did not act on the basis of such narrow self‐interest, and the vast majority supported the lockdown (e.g., Duffy & Allington, 2020). What is more, perceived personal risk bears no relation to whether people adhere to government instructions: whether or not one identifies with the broader community and hence acts on the basis of the risks to the community as a whole is the key driver (Jackson *et al*., 2020).

So, getting people to think in collective rather than personal terms is critical to controlling the pandemic (Reicher & Drury, 2020). Or, in the rather more forceful terms of New York Governor Andrew Cuomo: ‘Yeah it's your life do whatever you want, but you are now responsible for my life. You have a responsibility to me. It's not just about you … we started saying, “it's not about me it's about we.” Get your head around the we concept. It's not all about you. It's about me too. It's about we’.

#### How can we nurture the development and persistence of mutual aid and pro‐social behaviour?

The significance of such ‘we‐thinking’ is not limited to issues of adherence and social responsibility. The literature on behaviour in disasters and emergencies (Drury, 2018) suggests that the experience of common fate in such events leads to a sense of shared social identity that in turn underpins solidarity and cohesiveness between people – even strangers. We have seen numerous examples of ‘we‐thinking’ in the time of pandemic, which have played a key role in sustaining people through difficult circumstances. These range from neighbours knocking on doors to see whether people need help to over three million people contributing to more than four thousand mutual aid groups across the UK (Butler, 2020). So, how can we nurture such we‐thinking in order to build mutual aid in communities and ensure it endures even after the acute phase of the COVID‐19 pandemic is over?

#### What is the relationship between group membership, connectedness, and well‐being?

There is growing evidence of the role of group membership in sustaining both physical and mental health (Haslam, Jetten, Cruwys, Dingle, & Haslam, 2018). In addition to asking in general terms about how group identities are created, sustained, or else undermined in times of crises, we also need to investigate further the interface between group processes and health during and after periods of crisis. In other words: How can we keep people psychologically together even when they are physically apart and what is the relationship between face‐to‐face and virtual groups in terms of their health effects? More generally, can we learn from this in order to improve the plight of socially isolated people as we emerge from the acute phase of the pandemic?

#### Under what conditions does unity and social solidarity give way to intergroup division and social conflict?

Finally, in addressing the positive potential of social psychological processes, we must not forget their darker side. ‘We’ thinking can all too easily slip into ‘we and they’ thinking, where particular groups are excluded from the community and then blamed – even attacked – for the crisis. Thus, UN Head Antonio Guterres has warned of a ‘tsunami of hate’ unleashed by the pandemic (Davidson, 2020). This hate and violence can take different forms: of anti‐authority riots as in France (Willsher & Harrap, 2020), or of racist violence against minorities as in India (Mazumdaru, 2020).

In sum, insights from social psychology can be a valuable resource in a crisis; it can bring people together and generate constructive social power. But equally, it can set people apart and create problems that endure well beyond the crisis itself. It is evidently of the greatest importance to understand the processes that determine whether people unite or divide in hard times – and notably to understand the role of leadership, which has been so significant and so diverse in different countries during COVID‐19.

### Work environment and working arrangements

Consistent with previous pandemics (e.g., Rubin *et al*., 2010), the work‐related challenges of the pandemic have been particularly high and widely recognized for health and social care workers in direct contact with patients suffering the effects of COVID‐19, leaving them vulnerable to trauma, fatigue, and other manifestations of chronic stress. What is unique about COVID‐19 is that changed working conditions and anxiety about infection have affected almost all employees, with particular challenges being faced by delivery workers, shop assistants, teachers, emergency services personnel, care home staff, transport staff, and social workers.

The full economic severity of the COVID‐related restrictions is uncertain, although up to two million people could lose their employment in the UK alone (Wilson, Cockett, Papoutsaki, & Takala, 2020). For those people still working, and those about to return to work, there are notable changes that will likely affect working practices in the foreseeable future. Therefore, understanding the impact of the COVID‐19 pandemic on the work environment and new working arrangements is paramount to kick starting the economy and adjusting to daily life.

#### What is the impact of remote and flexible working arrangements on employee health, mental well‐being, teamwork, performance, organizational productivity, and colleague/client relationships?

For many workers, particularly those in white‐collar occupations, work took place entirely from home during the lockdown. It is possible that the lockdown will accelerate the general increase observed in home working practices (ONS, 2019). A move to greater levels of remote working has clear economic benefits for employers (e.g., reduced estates costs). The flexibility to balance work and family life is also attractive to many employees (cf. Strategic Review of Health Inequalities in England, 2010 ). Overall, the evidence points to positive benefits of remote working in terms of well‐being (Charalampous, Grant, Tramontano, & Michailidis, 2019), although these effects are not consistent. For example, it may lead to greater levels of professional isolation (Golden, Veiga, & Dino, 2008).

An increase in remote working will likely occur with a concomitant increase in the use of online technology to support communication and aspects of collaborative working. This has the potential to blur boundaries between work and home domains, resulting in negative impacts on well‐being and productivity from work–home interference (Van Hoof, Geurts, Kompier, & Taris, 2006). Greater use of technology may also be associated with different perceptual and cognitive demands that may affect productivity and well‐being including social connections with work colleagues (e.g., Mark *et al*., 2016).

#### What is the impact of physical distancing in the workplace on employee health, mental well‐being, teamwork, performance, organizational productivity, and colleague/client relationships?

Until an effective vaccine is available, physical distancing rules will need to continue to be in place in work environments and we may experience multiple stay‐at‐home versus return‐to‐work cycles. There is very little research exploring physical distancing and its effect on the general workplace, but returning to work will likely be both a welcome change and a potential stressor. While we have research from teams working in difficult and extreme environments (Power, 2018; Smith, Kinnafick, & Saunders, 2017) and research on professional isolation (Golden *et al*., 2008), this is an unprecedented opportunity to study adaptation across a breadth of individuals and organizational settings.

#### How can organizational resilience be developed to deal with the impact of COVID‐19 whilst supporting employees and protecting jobs?

The unprecedented demands that the pandemic has placed on organizations also offer a unique opportunity to understand how organizational resilience and preparedness for dealing with disruptions and emergencies can be developed. While a pandemic of this nature is rare, we can anticipate increasing periods of disruption due to COVID‐19 flare‐ups and additionally, for example, in response to climate‐induced events (e.g., recent Australian Fires, UK Flooding), which are predicted to occur more frequently (Banholzer, Kossin, & Donner, 2014). Although we know a lot about individual resilience, we know relatively little about organizational resilience, especially in the context of well‐being and performance (Taylor, Dollard, Clark, Dormann, & Bakker, 2019; Fasey, Sarkar, Wagstaff & Johnston, under review) and the ingredients such as the structures, processes, culture, and leadership that are essential for developing organizational resilience.

### Children and families

Parenting can be a challenging and anxiety‐provoking experience at any time, but the COVID‐19 pandemic has brought these challenges and anxieties into sharp focus. For most families, the lockdown will represent the longest period of parenting they have experienced without (1) the support of extended family members, friends, and childcare professionals; (2) the routine of school and out‐of‐school activities; and (3) any face‐to‐face social life outside the home. These changes in the social environment may have both negative and positive impacts on children and their families. At the most extreme end of the spectrum, the restrictions in place to combat the spread of the virus have been associated with worrying increases in domestic violence and child abuse. However, all families are likely to have experienced greater levels of stress (Social Care Institute for Excellence, 2020). The majority of carers with school‐age children are dealing with homeschooling for the first time, and many carers are having to adapt to working from home while also looking after their children and older relatives. These pressures will be particularly acute for single‐carer families. Of course, such multi‐tasking concerns apply only to carers fortunate enough to have maintained employment. It is important to support families during the current crisis, but also to understand the implications of these unprecedented changes in family life for family functioning and children's development as we emerge from the pandemic.

#### How will the COVID‐19 pandemic affect family functioning?

Many effects of the pandemic on children's development are likely to be indirect, functioning through its impact on caregiving and family functioning. It is crucial for this research to include family members such as grandparents and non‐resident parents and siblings. Children in families who are already vulnerable due to domestic violence or abuse, social or economic disadvantage, or physical or mental ill health are likely to be most adversely affected. There is an urgent need for research to examine how these vulnerabilities moderate changes in family functioning post‐pandemic and their impacts on the child.

The ability to regulate behaviour and emotional responses is a key aspect of successful social interaction in individuals of all ages (e.g., Baumeister & Heatherton, 1996; Kochanska, Murray, & Harlan, 2000). Family members may develop new self‐regulation strategies as a result of having extended contact with the same restricted group of people. While such strategies may be adaptive, individuals facing extreme social or financial challenges may cope by psychologically distancing themselves from family members, ruminating on negative events, or engaging in behaviours that are harmful. Understanding how adaptive and maladaptive self‐regulation strategies change post‐pandemic may prove useful in identifying individuals who need additional psychological support.

School closures and social restrictions may provide a unique opportunity for family members to gain insight into each other's lives, potentially reducing disagreements and improving family functioning. Research should investigate whether reporting such improvement during the crisis is associated with lower caregiving stress and better mental health. It is also important to study how families can maintain any positive aspects of functioning that have resulted from the pandemic as restrictions are eased.

#### How will the COVID‐19 pandemic affect children's development?

The effects of the pandemic will undoubtedly vary as a function of the child's age. While carers with young infants may have concerns about the negative impact of the lockdown on their babies' development, the infants themselves will be unaware of the abnormal nature of their social environment. Optimal later development is predicted by caregivers' ability in the first year of life to see the world from the infant's point of view and respond appropriately to their cues (e.g., Fraley, Roisman, & Haltigan, 2013; Zeegers, Colonnesi, Stams, & Meins, 2017). The social restrictions do not obviously impede this type of infant–caregiver engagement, and young infants may therefore be least affected by the pandemic. Older children who recognize the drastic changes in social contact may find transitioning back to pre‐pandemic social behaviour difficult. It is therefore important to study how children and young people manage this transition and investigate whether the lockdown has raised the incidence of emotional and behavioural difficulties. Studying the effects of the pandemic and its aftermath on particular groups that are known to be vulnerable to educational and health disadvantage (e.g., looked after children or children with developmental disorders) should be prioritized.

Positive effects of the pandemic on children's behaviour and social interaction are also anticipated. Many children and young people will have found new ways to communicate with friends, entertain themselves, and keep themselves physically active. Time away from school may have been spent learning new skills, developing new hobbies, or helping or supporting others. Investigating changes in children and young people's empathy, altruism, theory of mind, creativity, innovation, problem‐solving, and cognitive flexibility post‐pandemic will help shed light on potential positive outcomes of the social restrictions associated with the pandemic.

### Educational practices

The challenges posed by the COVID‐19 pandemic have never been more evident than for the education and well‐being of children and young people. In April 2020, a third of the world's population were experiencing extended periods of lockdown with closure of schools and nurseries. Parents, many of whom had work and other family responsibilities had to adopt the additional role of educator in home environments not set up for formalized learning. Ad hoc arrangements were put in place at speed by schools with limited opportunities to develop clear definitions of learning activities, provide access to learning resources, and establish effective home–school communication. Early surveys have shown wide variation in homeschooling arrangements, including stark differences between state and private schools in access to online learning and pupil–teacher communication (Sutton Trust, 2020).

There is a wealth of evidence about the factors that facilitate effective learning in schools, such as curricula and teaching strategies (Hattie, 2009). Other studies have established that children's academic attainment and adjustment are predicted by higher caregiver education (Erola, Janolen, & Lehti, 2016) and engagement in schooling (Harris & Goodall, 2008). However, little is known on how to set up and deliver home education effectively under the unique conditions of the pandemic. While for some children the extended period at home is likely to have distinct positive benefits, research prior to COVID‐19 on substantial externally driven disruptions in schooling has shown adverse effects on child achievement and well‐being (Meyers & Thomasson, 2017; Sunderman & Payne, 2009). The outcomes for the individual child are likely to depend on the capacity of families to step in and effectively support curriculum delivery at home.

Studies of other severe unplanned disruptions to schooling and family lives such as long‐running strikes and natural disasters have shown greatest impacts on long‐term educational and emotional outcomes for the most disadvantaged children (Jaume & Willén, 2019; Masten & Osofsky, 2010). At particular risk of disproportionate adverse outcomes are children from families living in poverty, those receiving social care support, individuals with special educational needs and disabilities, and young people with mental health problems. There are high levels of concern that the recognized attainment gap for children from disadvantaged families (Education in England: Annual Report 2019. Education Policy Institute) could be magnified by the pandemic conditions.

There is an urgent need to identify and understand both the positive and negative factors that influence children's educational outcomes during and after the pandemic, and to use this knowledge to target support to those who need it most. The unanticipated consequences of the pandemic pose challenges for conventional designs depending on pre‐intervention assessments. Understanding its impacts on children's lives will require a robust body of research that draws on the diverse research methods of psychological science. This will require large‐scale multidisciplinary data collection in addition to smaller‐scale quantitative and qualitative approaches that will be vital for understanding the experiences of children, families, and professionals. Some key questions to be addressed by this research are outlined below.

#### How do school closures influence children's educational progress and well‐being?

In addition to collecting data on home‐based support for learning, detailed contextual data are needed about social and environmental factors that are likely to interact in determining positive educational outcomes at particular educational phases (e.g., reading, writing, and maths in primary schools), as well as a range of mental health outcomes (e.g., anxiety, depression, self‐harm, resilience). This will include research into the effect of social distancing on a range of social outcomes in children and young people (e.g., inclusion/exclusion, friendships).

#### What kinds of support improve long‐term outcomes for children and young people?

Knowledge about the impacts of school disruptions on all children and young people will allow evidence‐based interventions and resources to be targeted at those with greatest need. Robust evaluations are required to scrutinize how interventions are accessed, by whom and with what degree of success.

#### How can support services be effectively delivered to vulnerable children and young people, families, and schools?

With reduced resources and restricted movement, professionals (such as practitioner psychologists) have had to adapt and develop new ways of delivering services. Researchers in psychological science have a key role to play in working with practitioners and service providers to evaluate systems put in place for monitoring and delivering professional support during and in the aftermath of the pandemic.

### Mental health

#### What are the immediate and longer‐term consequences of COVID‐19 for mental health outcomes?

There is expected to be an increase in mental health problems as a result of the COVID‐19 pandemic and the measures used to counter it. We already have evidence for the long‐term mental health effects of previous pandemics and disasters (e.g., Tam *et al*., 2004; Thompson *et al*., 2017; Wu *et al*., 2009) and an emerging literature on the near‐term effects of COVID‐19 (e.g., Ahmad & Rathore, 2020; Williamson *et al*., 2020). But previous pandemics have been more localized and circumscribed making COVID‐19 different. Social distancing, school closures, self‐isolation, and quarantine have lasted longer than anything previously experienced. We know that these factors, together with financial uncertainty and concerns about health, are predictive of mental health difficulties, particularly anxiety. The current pandemic amplifies these factors and not only exacerbates problems in those with pre‐existing mental health difficulties, but also increases the chance of new onset in those with no previous contact with mental health services. Concerns about mental health effects may be particularly heightened for children, who have experienced high levels of disruption to normative developmental opportunities (including opportunities for social and outdoor play) and education, and potentially high levels of family stress (https://emergingminds.org.uk/cospace‐study‐2nd‐update/).

Various poor mental health outcomes are also potentially associated with the disease itself. Information about the long‐term consequences comes from similar viruses such as SARS and the MERS. For example, many people who suffered from SARS seemed to experience detrimental psychological effects even a year later (Rogers *et al*., 2020; Tam *et al*., 2004; Thompson *et al*., 2017; Wu *et al*., 2009). Therefore, we need to establish the immediate and long‐term consequences of COVID‐19 on mental health outcomes in the population generally, but also in vulnerable, shielding, and self‐isolating groups (Box 1). We urgently need to understand how all these factors interact and whether these consequences will require psychological interventions and supports not currently available.

#### What changes in approaches resulting from the pandemic need to be harnessed for the future?

Even if the mental health consequences of this pandemic are not as predicted, we still expect increases in mental health problems. We know that mental health accounts for an increasing proportion of sick leave and that one in eight children and young people experience a diagnosable mental health problem (NHS Digital, 2018). Childhood mental health problems often recur in adulthood (Kessler *et al*., 2007) and are associated with physical health difficulties, poor academic, and occupational functioning, and are the primary predictor of low adult life satisfaction (Layard, Clark, Cornaglia, Powdthavee, & Vernoit, 2014). The increased prevalence will place a further burden on a mental health system that was already stretched and will increase waiting times and accentuate gaps in care. During the pandemic, mental health services rapidly changed. Inpatients were discharged, even if they were detained in hospital because they were a risk to themselves or others. Some people benefited, but we do not know how this reduction in bed use was managed. Was it because the right supports and accommodation were provided?

The move to remote contact in mental health services had been slow and of varied quality prior to COVID‐19 with challenges for both staff and service users. But the shift during the pandemic was swift, and although undoubtedly NHS staff felt pressure during the changeover, there now seems to be a steadier state. Again, some service users may have benefited from this change with reductions in travel and, for some, better access to care and treatment. However, although the digital divide is reducing (Robotham, Satkunanathan, Doughty, & Wykes, 2016), it remains highest in those who already have high unmet needs, including people in rural areas, those on lower incomes, people with lower levels of formal education, and older people. If remote working is to be a beneficial part of an evolved mental health service, then we need to understand how to provide that ‘webside’ manner that will increase adherence and promote a therapeutic alliance. We also urgently need to evaluate the effectiveness of remotely delivered, digital interventions in the immediate and longer term. Future interventions will need to be deliverable remotely, depending on local resources. For example, from an international perspective, many low‐to‐middle‐income countries do not have high broadband penetration; hence, optimizing digital delivery that depends strongly on good internet connections will further widen the welfare gap.

### Physical health and the brain

The effects of COVID‐19 on health outcomes will be far‐reaching and complex. For those falling ill, there are the direct consequences of the disease symptoms, such as respiratory failure in severe cases, alongside potentially direct viral effects on the brain. There are also more indirect population‐wide effects of COVID‐19 pandemic‐related stress and anxiety on physical and mental health, not only from the disease itself but also from changes in lifestyle including delayed treatment and screening for other known or suspected conditions. Moreover, it is also likely that from an international perspective, in many low‐to‐middle‐income countries, the pandemic will result in greater hunger/starvation, which will have severe impacts upon health.

#### Does COVID‐19 have neurological effects on the brain with consequences for mental health?

At one level, COVID‐19 might alter mental health by the direct actions of the specific virus (severe acute respiratory syndrome coronavirus‐2; SARS‐CoV‐2) on the brain. While neurological dysfunction is often described in COVID‐19, including dizziness, and loss of taste and smell, these conditions are common to other respiratory tract infections and need not reflect a neurological disease *per se* (Needham, Chou, Coles, & Menon, 2020). Data from cerebrospinal fluid and post‐mortem analyses will help resolve issues over the penetrance of SARS‐CoV‐2. It is, however, known that the target receptor for SARS‐CoV‐2 is the angiotensin‐converting enzyme‐2 receptor (ACE 2). Disruption of the blood–brain barrier during illness might enable entry of the virus, potentially aided by the presence of ACE 2 receptors in glial cells and brain endothelium. Other potential routes of entry include the cribriform plate and olfactory epithelium, as well as via peripheral nerve terminals, permitting entry to the CNS through synapse connected routes (Ahmad & Rathore, 2020).

At the same time, there is an array of immunological responses, including the cytokine ‘storm’ in severe cases, alongside non‐immunological insults to the central nervous system provoked by COVID‐19. The latter include hypoxia, hypotension, kidney failure, and thrombotic and homeostatic changes involving neuroendocrine function (Needham *et al*., 2020). Together and separately, they may contribute to brain dysfunction in ways that vary with the severity of the infection, other underlying conditions (Needham *et al*., 2020), and the treatment for those other conditions (South, Diz, & Chappell, 2020). Large‐scale studies help confirm differential clinical risk factors for death following infection (Williamson *et al*., 2020), prompting genotype analyses, while noting that COVID‐19 might also induce epigenetic changes, including ACE 2 demethylation (Sawalha, Zhao, Coit, & Lu, 2020). Additional health concerns include post‐viral fatigue and whether it might provoke a long‐lasting syndrome.

Research consortia are initiating comparisons between populations that have or have not contracted COVID‐19. Challenges for psychological scientists include how to assess impacts on cognition and mental health, both in the short term and long term. A part of this challenge is how to deliver effective, online psychological testing (e.g., for ‘shielded’ populations), or to help follow‐up large population cohorts, while not biasing the sample away from those least likely to use these platforms. An integral part of some investigations will be the inclusion of multiple neuroimaging methods, despite the era of distancing. Just one of many questions would be the impact of COVID‐19 on mild cognitive impairment and its conversion to dementia. There is a premium on studying pre‐existing cohorts (e.g., UK Biobank, ALSPAC), where retrospective, baseline data exist. Such data are especially precious in the present landscape where everyone is, to some degree, affected by the pandemic. The power of these pre‐existing cohorts will, however, be heavily influenced by the proportion of the population who contract COVID‐19.

#### What are the psychobiological impacts of the COVID‐19 pandemic on physical and mental health?

Despite the umbrella term ‘stress’ covering many different things, there is agreement that in its different forms, stress can lead to physiological changes (e.g., neuroendocrine, cardiovascular), with negative consequences for health (O'Connor, Thayer & Vedhara, in press). Three principal research questions can be identified: (1) To what extent does pandemic‐related stress, anxiety, and worry impact on biological mechanisms that influence health (i.e., hypothalamic–pituitary–adrenal axis regulation and cortisol dynamics, the autonomic nervous system, and gene expression) as well as on health behaviours (e.g., eating, sleep, alcohol consumption)? (2) How best to counter their adverse effects? and (3) How might such stress exacerbate existing medical and mental health conditions, and for how long? For all three questions, there will be considerable variations between groups and individuals (Box 1). One challenge will be to collate and verify relevant information, including that from ‘smart’ devices that can provide daily physiological data, activity information, and other measures of diurnal patterns, including sleep.

One of the groups most likely to be negatively affected by stress is health care professionals. The pandemic may exacerbate the already high prevalence of secondary traumatic stress, burnout, and physical exhaustion among health care professionals, as well as impact on patient safety and medical error (e.g., Dar & Iqbal, 2020; Figley, 1995; Hall, Johnson, Watt, Tsipa, & O'Connor, 2016), due to excessive workload and workplace trauma (e.g., Itzhaki *et al*., 2018). While resources such as support from managers and colleagues can help protect health care professionals against traumatic stress, the longer‐term impact is likely to be substantial on individuals, their families, on the national health services and the wider care industry. Amongst other groups of concern (Box 2) are those caring for a vulnerable relative or partner at home.

### Behaviour change and adherence

One novel feature of daily life in the wake of the COVID‐19 pandemic in countries around the world are near‐daily government briefings. One focus of these briefings is government instructions to the public as to how to behave. Adherence to these and future instructions will be key to dealing with future crises. Moreover, many sections above share in common the requirement that people adhere to instructions, whether it is practitioners delivering psychological therapies effectively over the telephone or employees continuing to maintain physical distancing at work. In the initial response to the pandemic, many governments instructed people to (1) stay inside as much as possible; (2) stay >1 m away from other people at all times; and (3) maintain hand hygiene, among other measures such as wearing face coverings. The evidence suggests that public adherence to government COVID‐19‐related instructions worldwide has been high (ONS, 2020), but it is not clear for how long people will continue to adhere to instructions that impinge on personal freedoms. What is clear is that there is a dearth of workers sufficiently trained to advise policymakers and to implement behaviour change interventions rapidly and at scale. The British Psychological Society's guidance on behaviour change is a good starting point for ensuring that instructions and messaging is clear (British Psychological Society, 2020a). Appointing chief behavioural science advisers to governments would ensure that cutting‐edge psychological science advice is placed at the heart of policymaking.

As people begin to emerge from the acute phase of the pandemic and the changes that were made to tackle it, it is important that psychological science is at the heart of ensuring that health‐enhancing behaviours are sustained and that health‐damaging behaviours are changed or prevented. There are numerous approaches to developing such interventions, including the behaviour change wheel (Michie, Atkins, & West, 2014) and intervention mapping (Bartholomew Eldrigde *et al*., 2016), but they require the expertise of psychological scientists to deliver and to evaluate them (West, Michie, Rubin, & Amlôt, 2020). One of the main challenges now, and in the future, will be to ensure there is a workforce equipped with the competencies to develop behaviour change theory and tools that will bring about sustained changes in behaviour. Taught post‐graduate courses exist that could be scaled up and/or adapted to continuing professional development qualifications to meet this demand and help ensure that the changes in behaviour that will be required for the foreseeable future are sustained.

#### How do we best apply existing theories and tools to promote sustained behaviour change among policymakers, key workers, and the public/patients?

We sometimes forget that we have the theories and evidence for solutions that can be applied at pace to address novel problems. Although we have never seen a lockdown before and so cannot predict what the outcomes will be directly, we do know what processes underpin adherence to instructions, and so can advise on the levers that can sustain adherence. In unprecedented and uncertain times now and in whatever the future might bring, the nature of psychological science allows us to make unique and invaluable contributions. If the COVID‐19 pandemic teaches us one thing, it is on the need to accelerate the translation of evidence from psychological science into practice.

#### How do we develop new theories and tools to promote sustained behaviour change?

At the same time, we should not forget the ‘slow’ approach to research (Armitage, 2015) that involves addressing key research questions with multiple perspectives and methodologies, and accumulating such knowledge in PRISMA‐guided systematic reviews. It is vital that continued investment is made into behaviour change research. Only with this can we refine and develop the theories that best explain human behaviour (e.g., Michie *et al*., 2014). Key research priorities include identifying which behaviour change techniques work best, for whom, in which contexts, and delivered by what means (e.g., Epton, Currie, & Armitage, 2017) as well as how to counter the conspiracy theories and misinformation that arise during crises that seem to be aimed at derailing the very behaviours required to keep us safe and to reduce risk.

## Call to action

In this position paper, we have set out seven research priority domains in which psychological science, its methods, approaches, and interventions can be harnessed in order to help governments, policymakers, national health services, education sectors, economies, individuals, and families recover from COVID‐19. These are mental health, behaviour change and adherence, work, education, children and families, physical health and the brain, and social cohesion and connectedness. We have also highlighted that a clear overarching research priority relates to understanding the inequalities in the effects of the pandemic and recovery; recognizing the vulnerability and resilience factors that will be key to understanding how the current pandemic can inform and prepare us for dealing with future crises. We call on psychological scientists to work collaboratively with other scientists in order to address the research questions outlined, refine them and to adopt multidisciplinary working practices that combine different disciplinary approaches. An important next step will be to engage with wider stakeholders, potential users, individuals with lived experience, and beneficiaries of the research. Addressing each of the research priority domains will benefit enormously from larger scale working and coordinated data collection techniques and the establishment of research consortia with their associated economies of scale. We also call on psychological scientists to further develop and adapt innovative research methodologies (e.g., remote testing and intervention delivery, online data collection techniques), while maintaining high‐quality, open, and rigorous research and ethical standards in order to help with the recovery as we emerge from the acute phase of the crisis.

## Conflicts of interest

All authors declare no conflict of interest.

## Author contributions

Daryl B. O'Connor and Christopher J. Armitage (Conceptualization; Methodology; Writing – original draft; Writing – review & editing), John Aggleton, Cary L. Cooper, Susan Gathercole, Sandra Dunsmuir, Elizabeth Meins, Stephen D. Reicher, Til Wykes, Lisa J. Morrison Coulthard and Debra Malpass (Methodology; Writing – original draft; Writing – review & editing). Bhismadev Chakrabarti, Cathy Creswell, Susan T. Fiske, Brendan Gough, Jane L. Ireland, Marc V. Jones, Adam Jowett, Carolyn Kagan, Maria Karanika‐Murray, Linda K. Kaye, Veena Kumari, Stephan Lewandowsky, Stafford Lightman, B. Paul Morgan, Daniel L. Schacter, Susan M. Sherman, Victoria Simms, Antony Williams (Review & editing).

## Footnotes

^1^
The Expert Group consisted of Daryl B. O’Connor (Chair), Christopher J. Armitage (co‐Chair), John Aggleton, Cary L. Cooper, Susan Gathercole, Sandra Dunsmuir, Elizabeth Meins, Stephen D. Reicher, Til Wykes, supported by Lisa Morrison Coulthard and Debra Malpass.

^2^
The Advisory Group consisted of Bhismadev Chakrabarti, Cathy Creswell, Susan T. Fiske, Brendan Gough, Jane L. Ireland, Marc V. Jones, Adam Jowett, Carolyn Kagan, Maria Karanika‐Murray, Linda K. Kaye, Veena Kumari, Stephan Lewandowsky, Stafford Lightman, B. Paul Morgan, Daniel L. Schacter, Susan M. Sherman, Victoria Simms, Antony Williams.

## Final 40 Research Priorities included in survey of psychological scientists

**Table:**

The set of priorities utilized for the survey of the psychological community. How do we increase adherence (and ability to adhere) to UK government COVID‐19‐related instructions? How do we promote maintenance of positive behaviour changes and reverse negative behaviour changes resulting from COVID‐19‐related lockdown? How do we address the negative psychological impacts of the COVID‐19 pandemic? How do we maximize recovery from COVID‐19 for those infected with the virus? What is the impact of COVID‐19‐related stress on biological processes and health outcomes? What makes people adhere to anti‐COVID measures? What are the bases of anti‐social behaviours such as stockpiling? How do mutual aid groups form and what makes them endure? When does social cohesion give way to scapegoating, prejudice, and intergroup conflict? What creates (or prevents) the potential for protests and collective disorder in the crisis? What are the long‐term mental health effects of COVID‐19? What coping mechanisms are useful in reducing mental health problems during a pandemic? How do we provide beneficial remote psychological therapy and maintain therapeutic alliance? Has discussion of mental health during the pandemic reduced stigma and discrimination in the community? People detained in hospital under the Mental Health Act were discharged to free up beds – how was this possible? What are the impacts of COVID‐19 infection, treatment, and recovery on cognition, behaviour, and the brain? What are the drivers of COVID‐19‐related stress and its cognitive, neural, and physiological mechanisms and consequences? What are the perceptual and cognitive demands of digital and other alternative forms of communication and how do they impact on work and social connectivity? What factors influence the effectiveness of communication of scientific evidence and national guidance, and how do they influence behaviour? How do restrictions of movement, communication, and social support influence the cognitive, physical, and mental health of older individuals, and what factors lead to improved outcomes? How has the COVID‐19 pandemic affected parenting? How has the COVID‐19 pandemic affected children's development? How has the COVID‐19 pandemic affected family functioning? Which factors moderate family members' response to the COVID‐19 pandemic? What support is most effective for families during the COVID‐19 pandemic? How do we assess biological markers of health and well‐being remotely? How can we use biological markers to facilitate people's return to work? How do we link COVID‐19‐related biomarkers to existing population cohort databases? How do we address the negative biological impacts of the COVID‐19 virus on mental health? What are the impacts of COVID‐19 infection, treatment, and recovery on the brain? How do school closures influence educational progress, and physical and mental health outcomes for all children and young people? What ‘homeschooling’ practices are associated with positive educational and psychological outcomes? What is the effect of social distancing on a range of social outcomes in children and young people? What methods are used to track, monitor, and deliver local authority support services to vulnerable children and young people, families, and schools during lockdown, at transition back to school, and after return to school? How are educational and psychological interventions allocated, structured, delivered, and evaluated for children and young people in need, after schools have reopened? What is the impact of remote and flexible working arrangements on employee health, mental well‐being, teamwork, performance, organizational productivity, and colleague/client relationships? What is the impact of social distancing in the workplace on employee health, mental well‐being, teamwork, performance, organizational productivity, and colleague/client relationships? What managerial behaviours are most effective to manage remote working, possible mental health issues, job insecurity, and productivity? What is the risk of longer‐term mental ill health among frontline staff after the immediate crisis? How can organizational resilience be developed to deal with the impact of COVID‐19 whilst supporting employees and protecting jobs?
